# An Uncommon Presentation of Cryptococcal Meningitis in an Immunocompetent Patient: A Case Report

**DOI:** 10.5811/cpcem.2021.8.53368

**Published:** 2021-10-12

**Authors:** Kelly Correa, Scott Craver, Amar Sandhu

**Affiliations:** * Henry Ford Wyandotte Hospital, Department of Emergency Medicine and Graduate Medical Education, Wyandotte, Michigan; † Ascension St. Vincent Indianapolis Hospital, Department of Emergency Medicine, Indianapolis, Indiana; ‡ Henry Ford Wyandotte Hospital, Department of Emergency Medicine, Wyandotte, Michigan

**Keywords:** case report, immunocompetent, cryptococcal meningitis, altered mental status, headache

## Abstract

**Introduction:**

Meningitis is a serious and potentially life-threatening infection of the central nervous system. *Cryptococcus neoformans* is a rare fungal cause of meningitis that commonly presents with atypical symptoms. Although this infection is most common in immunocompromised patients, it also occurs in immunocompetent patients. This case report describes an atypical presentation of cryptococcal meningitis in a seemingly immunocompetent patient.

**Case Report:**

A 40-year-old immunocompetent patient with no significant past medical history had visited the emergency department (ED) five times within a span of 30 days reporting dental pain and headache. Throughout each of the visits, no clear symptoms signaling the need for a meningitis workup were observed, as the patient had been afebrile, displayed no nuchal rigidity, and his presenting symptoms subsided within the ED after treatment. A lumbar puncture was performed after emergency medical services brought the patient in for his sixth ED visit, initially for stroke-like symptoms and altered mental status. Spinal fluid was indicative of cryptococcal meningitis.

**Conclusion:**

This case highlights the challenge of identifying cryptococcal meningitis in the ED, particularly in immunocompetent patients who do not display classic meningitis symptoms. It also highlights the importance of keeping a broad differential and carefully ruling out diagnoses when patients return to the ED multiple times for the same complaint.

## INTRODUCTION

*Cryptococcus neoformans* (*C. neoformans*) mediated meningitis is a common opportunistic infection in immunocompromised patients, many of whom are positive for human immunodeficiency virus (HIV).^1^ Other susceptible individuals include those undergoing cancer treatment or taking immunosuppressive medications for transplants or autoimmune diseases. Pertinent symptoms of cryptococcal meningitis include fever, headache, nuchal rigidity, and new onset altered mental status. If the disease is suspected, the patient should undergo imaging of the brain (computed tomography or magnetic resonance imaging) and lumbar puncture.^1^ The estimated number of hospitalizations for cryptococcal meningitis in the United States (US) is roughly 3,400 cases per year, with 700 deaths annually in both immunocompromised and immunocompetent individuals, indicating a rather high mortality rate.^2^ Almost 22% of cryptococcal meningitis hospitalizations in the US in 2009 were in individuals without HIV.^3^

While the prevalence of cryptococcal meningitis in patients with HIV within the US has been declining, cryptococcal meningitis in immunocompetent and non-HIV infected patients has been more persistent, accounting for a substantial proportion of all cryptococcal meningitis cases.^4^ Identifying the true incidence of cryptococcal meningitis in immunocompetent patients is challenging, since non-HIV infected patients may have a range of levels of immunocompetence. One single-center study stratified patients into HIV-positive, organ transplant recipient, and non-HIV/non-organ transplant groups to better define the immunocompetent population and showed that of 302 cryptococcal meningitis cases, 36% were from the non-HIV/non-organ transplant group, which shows that most cases occurred in patients with a known immunocompromised status.^7^

Interestingly, cryptococcal meningitis has shown higher mortality rate in non-HIV infected individuals than in HIV-infected patients in the US (13.3% and 10.5%, respectively).^3^ Clinical presentations can vary, and classic symptoms of meningismus only occur in some patients. Immunocompetent patients may have a longer time from the onset of illness to presentation, a more evident inflammatory response (leading to elevated intracranial pressure), and various comorbidities that may also contribute to poor prognosis.^3^,^5^,^6^,^7^

The purpose of this case report is to illustrate how patients with cryptococcal meningitis may not have the risk factors, patient history, or physical exam findings that are commonly seen in meningitis. Additionally, we emphasize that immunocompetent patients are likely to develop cryptococcal meningitis in the absence of a classic meningitis presentation, thus, the disease should be considered in every patient who presents to the emergency department (ED) with headache, altered mental status, or behavioral change.

## CASE REPORT

A 40-year-old man was brought to the ED by emergency medical services after he was found outdoors displaying an altered mental status, right-sided facial droop, headache, and unsteady gait. On initial evaluation, the patient was drowsy, following commands poorly, and could not answer questions appropriately. His initial Glasgow Coma Scale score was 13 (eye - 3, motor - 4, verbal - 6). Provocative testing revealed inconsistent right-sided ptosis with an otherwise non-focal neurologic exam.

Given this presentation, there was a concern for stroke. The patient underwent computed tomography and computed tomography angiography of the head and neck; both were negative for any evidence of ischemic or hemorrhagic stroke or other abnormalities. During his continued evaluation, he reported a headache, which he described as left temporal pressure with associated dental pain. He reported exacerbation of the headache with light and sound but reported not having had any nausea or vomiting. He conveyed that his headache was consistent with previous migraines, just more severe. He did not report any neck stiffness, fever, or sweats. Medical records showed that the patient had been in the ED five other times in the past 30 days with either headache or dental pain. On one visit the patient reported dental pain, was noted to have poor dentition, and pain was improved after dental block. On another visit the patient reported chronic headaches that were relieved by anti-inflammatory medications. Each of these previous assessments did not reveal any fever, neck stiffness, or altered mental status and thus did not trigger concern for intracranial pathology or meningitis requiring further workup. Each time, after symptomatic improvement with medications given in the ED, the patient was discharged and instructed to follow up with his family medical doctor.

**CPC-EM Capsule**

What do we already know about this clinical entity?

*Cryptococcal meningitis is a rare form of fungal meningitis that is most common in immunocompromised individuals. It often presents with atypical symptoms and has a high mortality rate.*

What makes this presentation of disease reportable?

*This case of cryptococcal meningitis in a healthy patient without any identifiable risk factors reviews his atypical presentation, multiple visits, and the symptom that led to diagnosis.*

What is the major learning point?

*Cryptococcal meningitis can occur in immunocompetent patients. Physicians must have a high suspicion for this disease in patients presenting with change in behavior, and/or headaches.*

How might this improve emergency medicine practice?

*Knowledge of the possible atypical presentations of cryptococcal meningitis among physicians can lead to earlier diagnosis, treatment, and functional outcome in these patients.*

Later in the patient’s ED course, a family member arrived and reported that the patient had been mentally decompensating over the previous two weeks. The family member stated that the patient had been acting abnormally, including urinating, and defecating in his bedroom and walking around the house naked. The family member was not aware of the patient having had any recent illness, recent travel, exposure to birds, or having pets at home. The family member reported that the patient worked as a sandblaster and had a sporadic history of marijuana and alcohol use. Given the patient’s behavioral change, a lumbar puncture was performed for further investigation.

The procedure was performed in the standard fashion, positioned in the left lateral decubitus position. The initial opening pressure was 42 centimeters of water (cmH_2_O) (reference range: 5–25 cmH_2_O). Analysis of cerebrospinal fluid (CSF) from tube four revealed a red blood cell count of 5 millimeters cubed (mm^3^) (reference value: 0 mm^3^), white blood cell count of 178 mm^3^ (reference range: 0–5 mm^3^), neutrophils 40% (reference value: 0%), lymphocytes 35% (reference range: 60%–70%), eosinophils 4% (reference value: 0%), protein 100 milligrams per deciliter (mg/dL) (reference range: 15–55 mg/dL), glucose 20 mg/dL (reference range: 40–80 mg/dL) and lactic acid 5.4 millimoles per liter (mmol/L) (reference range: 1.2–2.4 mmol/L). Given the concern for meningitis, the treatment team started the patient on empiric antibiotics (vancomycin 2 grams, ceftriaxone 2 grams, and acyclovir 710mg given parenterally) while the patient was in the ED.

The patient was admitted to the hospital where he was followed by infectious disease specialists and given continued empiric medications as described above. Two days after the initial lumbar puncture, the cryptococcal CSF antigen test returned positive. The patient was then started on amphotericin 450mg parenterally and flucytosine 1750mg orally. Cerebrospinal fluid and blood cultures tested positive for *C. neoformans* on day three. The patient received six therapeutic lumbar punctures during his admission for increased intracranial pressure, as well as eventual placement of a ventriculoperitoneal shunt for persistently elevated intracranial pressure after two months of therapy. A fourth-generation antigen/antibody HIV enzyme-linked immunosorbent assay (ELISA) test during admission was negative. Investigation and workup for other disease processes causing immunosuppression (including cirrhosis, autoimmune disorders, hematologic malignancy, sarcoidosis, previous steroid use, immunosuppressive therapy) were all negative. Further exploration into his alcohol use noted that it was “sporadic” and was thought to be non-contributory. Magnetic resonance imaging done during his hospitalization revealed increased T2/weighted-fluid-attenuated inversion recovery signal representing ventriculitis consistent with cryptococcal meningitis (Image). Hallucinations and odd behaviors were continually noted during the patient’s admission, although these symptoms improved gradually. Upon discharge from the hospital, the patient was placed on 1600mg of oral fluconazole daily for three months and then continued maintenance therapy of fluconazole 400mg for 12 months. One year from the patient’s initial diagnosis of cryptococcal meningitis, he had some improvement in his cognition and was able to live independently, though he continued to struggle with symptoms of headache despite having a ventriculoperitoneal shunt and was unable to work. He continues to follow with infectious disease to ensure continued remission from cryptococcal meningitis.

## DISCUSSION

The differential diagnosis of headaches and altered mental status is broad and requires thoughtful consideration when narrowing down the etiology of a patient’s symptoms. The case presented here illustrates how an otherwise healthy, immunocompetent individual may present with seemingly mild symptoms, such as headache, before more serious symptoms of cryptococcal meningitis develop, such as altered mental status. Our patient’s experience highlights the challenge of identifying cryptococcal meningitis in the ED since individuals may not present with obvious signs of meningismus and confirmatory diagnosis via antigen testing takes time and is unlikely to be available while the patient is in the emergency department.

Cryptococcal meningitis is atypical in otherwise immunocompetent patients, with only 0.4 to 1.3 cases per 100,000 people in the United States.^8^ Studies have shown that *C. neoformans* uses its many virulence factors and phenotypic plasticity to avoid host macrophages after inhalation from the environment, allowing it to bypass the blood-brain barrier and multiply within a nutrient-depleted environment.^9^ There are two leading causes of cryptococcal meningitis infection. The first is a high level of organism exposure, such as exposure to bird excrement where *C. neoformans* are found.^10^ The second is immunosuppression from conditions such as HIV, alcoholism, diabetes mellitus, or autoimmune disease.^11^

The current criteria used to evaluate a patient’s risk of having cryptococcal meningitis is suboptimal. Meningismus, a classic finding in meningitis, is defined as neck rigidity, photophobia, and headache; however, this constellation of symptoms occurs in less than 20% of patients with cryptococcal meningitis.^11^ Therefore, accurate diagnosis in the ED is challenging. Most patients with cryptococcal meningitis display at least one of the following symptoms: headache, altered mental status, nuchal rigidity, or fever; headache being the most commonly reported symptom.^11^,^12^ When patients who lack the obvious risk factors for cryptococcal meningitis present with vague symptoms or present multiple times with the same symptoms, such as headache in the case of our patient, the physician may mistakenly conclude that the patient has a recurring condition and not an acute pathology. Thus, anchoring bias is a particular barrier to swift and accurate diagnosis of cryptococcal meningitis in otherwise healthy, immunocompetent patients.

Management of cryptococcal meningitis after diagnosis starts with induction therapy to quickly reach sterilization of the CSF and normally includes intravenous combination antifungal therapy with amphotericin B and flucytosine.^13^ However, this decision should be made in consultation with an infectious disease specialist. Relieving elevated intracranial pressure via lumbar puncture (or VP shunt) until pressure normalizes is also an important component of treatment for cryptococcal meningitis, due to the significant inflammatory burden.^13^,^14^ A lumbar puncture is typically repeated after two weeks of antifungal induction therapy to confirm sterilization of the CSF, even among patients who have clinically improved.^13^ If the CSF is sterile, therapy can be de-escalated to a consolidation dosing range (400mg fluconazole daily). Consolidation and maintenance therapy with fluconazole can proceed for a year or more.^11^ Note that specific recommendations vary for specific populations, such as HIV-infected individuals, organ transplant recipients, children, and pregnant women.^14^ A comprehensive treatment of cryptococcal meningitis management for ED clinicians can be found in Fisher et al. 5

Patients with cryptococcal meningitis who are not presenting with classic signs and symptoms of meningitis and who do not have the main risk factors (immunosuppression), often have poor outcomes because diagnosis and treatment are delayed.^11^ The most important prognostic factors are the nature of the underlying immunosuppression and the concurrent disease processes. Other factors conferring poor prognosis include positive India ink examination of the CSF, CSF white blood cell count less than 20 μL, initial CSF or serum cryptococcal antigen titer greater than 1:32, and high opening pressure on lumbar puncture.^15^

## CONCLUSION

Overall, *C. neoformans* meningitis is a rare cause of meningitis in immunocompetent individuals; however, it is still important to consider in patients with headache and altered mental status given its insidious onset and high mortality rate. A careful and detailed history is warranted for every encounter to evaluate risk factors for serious diseases and narrow the differential diagnoses. Furthermore, when a patient presents multiple times to the ED with symptoms like headache, it is imperative that clinicians reconsider the differential diagnosis, initiate immediate testing for meningitis-causing microorganisms and begin appropriate supportive care.

## Figures and Tables

**Image:**
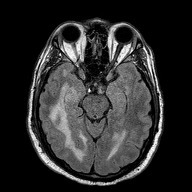
Magnetic resonance imaging without contrast. The hyperintense areas (black arrows) in the cerebrum are increased T2/weighted-fluid-attenuated inversion recovery signal representing ventriculitis consistent with cryptococcal meningitis.
